# A Possibility for Quantitative Detection of Mechanically-Induced Invisible Damage by Thermal Property Measurement via Entropy Generation for a Polymer Material

**DOI:** 10.3390/ma15030737

**Published:** 2022-01-19

**Authors:** Takenobu Sakai, Naohiro Takase, Yutaka Oya, Jun Koyanagi

**Affiliations:** 1 Graduate School of Science and Engineering, Saitama University, Shimo-Okubo, Sakura-ku, Saitama 338-8570, Japan; sakai@mech.saitama-u.ac.jp; 2 Department of Materials Science and Technology, Graduate School of Tokyo University of Science, 6-3-1 Niijuku, Katsushika-ku, Tokyo 125-8585, Japan; 8216044@alumni.tus.ac.jp; 3 Research Institute for Science and Technology, Tokyo University of Science, Tokyo 125-8585, Japan; oya@rs.tus.ac.jp; 4 Department of Materials Science and Technology, Tokyo University of Science, 6-3-1 Niijuku, Katsushika-ku, Tokyo 125-8585, Japan

**Keywords:** material damage, entropy generation, heat capacity, molecular dynamics simulation, differential scanning calorimetry

## Abstract

Entropy generation from a mechanical and thermal perspective are quantitatively compared via molecular dynamic (MD) simulations and mechanical and thermal experiments. The entropy generation values regarding mechanical tensile loading—which causes invisible damage—of the Polyamide 6 (PA6) material are discussed in this study. The entropy values measured mechanically and thermally in the MD simulation were similar. To verify this consistency, mechanical and thermal experiments for measuring entropy generation were conducted. The experimentally obtained mechanical entropy was slightly less than that calculated by MD simulation. The thermal capacity is estimated based on the specific heat capacity measured by differential scanning calorimetry (DSC), applying the assumed extrapolation methods. The estimated entropy generation was higher than the aforementioned values. There is a possibility that the entropy-estimating method used in this study was inappropriate, resulting in overestimations. In any case, it is verified that entropy increases with mechanical loading and material invisible damage can be qualitatively detected via thermal property measurements.

## 1. Introduction

Recently, polymers and their composites have been widely used in the aerospace, energy, and automobile industries, as well as various types of infrastructure. Accurately predicting their lifetimes is of great importance for these applications. To predict the lifetime of polymers and their composites, the time-temperature superposition principle (TTSP) is usually applied to the materials and long-term time-dependent behavior is predicted via experiments performed at high temperatures [1,2,3,4,5,6]. However, this method is inapplicable to previously used materials owing to their unknown histories, such as thermal and mechanical load histories. To understand the remaining lifetime of used materials and/or materials in use, their invisible damage, i.e., free volume change, nano-sized voids, and crack initiation, should be known. However, there is no method for measuring this. It is difficult to detect the extent to which damage is accumulated in an existing polymer material that looks almost intact.

These materials are damaged by external forces. In the case of polymeric materials, the free volume in the materials increases even under a small external force [7] and grows with additional external force, resulting in plastic deformation, voids, and cracks. In this situation, the entropy of the system increases [8]. Studies have described the failure criteria for solid materials based on irreversible entropy [9,10]. Naderi et al. revealed that failure occurs when entropy generation inside materials reaches a threshold value [9]. Sato et al. reported that the fracture entropy generation of TriA-X polyimide was ~15.5 kJ/m^3^K [11]. Entropy-based damage evaluation has been experimentally confirmed for metallic [12,13,14], polymeric [9], and composite materials [15,16,17,18]. In the following, we distinguish between thermal and mechanical entropies. Thermal entropy is defined by generated heat, estimated by a heat or energy balance from the first law of thermodynamics, while mechanical entropy is defined using viscous dissipation, calculated by subtracting the elastic energy from the strain energy.

Molecular dynamics simulations (MD) have become a useful tool for clarifying the causes of experimental phenomena [19,20,21,22,23,24,25,26,27,28,29,30,31,32]. Regarding the mechanical properties of polymers, Fan et al. predicted the thermomechanical properties of epoxy [19] and Fujimoto et al. investigated the impact fracture of poly-methyl-methacrylate (PMMA) and polycarbonate (PC) [20]. The difference between the experimental and numerical results originates from the scale difference in time and dimensions; however, the MD results and experimental results have recently increased in consistency [21]. MD simulations of the thermal properties of polymers and their composites were conducted by Chen et al. [22], Cheng et al. [23], and Bhowmik et al. [24]. Bhowmik et al. calculated the specific heat of polymers, which is related to thermodynamic entropy, via MD simulations. Takase et al. [25] analyzed the fracture entropy generation of polyamide 6 (PA6) using MD simulations and observed that the fracture entropy generation under multiaxial conditions was almost consistent. The entropy generation and free volume change were observed to depend on mechanical loading. Here, it is assumed that directly measuring the entropy will enable the remaining lifetime to be assessed based on invisible damage to the materials already in use; it is connected to the sustainable development goals (SDGs).

To measure the entropy directly, we focused on thermal entropy. Further mechanical loading is necessary to measure mechanical entropy generation, which is calculated via dissipated energy. Therefore, the practical application of this method to structural materials is difficult. However, if the values of mechanical entropy generation and thermal entropy generation are similar, they may inherently be the same and we can measure the entropy generation via thermal analysis, such as specific heat capacity measurements conducted using differential scanning calorimetry (DSC) [33]. In this study, the thermal entropy generation of PA6, with or without tensile loading, was measured using DSC and compared to the mechanical entropy generation obtained from the strain energy, which was calculated using data from experimental tensile tests and tensile analysis by MD.

## 2. MD Simulation

### 2.1. Polymer System

In the present study, tensile analysis using MD was performed on PA6 resin. The polymerization degree of PA6 was set at 30 using an all-atom model and a system with ~570,000 atoms was prepared. After the molecular chains were randomly arranged in the cell, the isothermal-isobaric (constant number of particles: N, pressure: P, temperature: T, as NPT) ensemble was used to achieve equilibrium at 650 °C above the melting point. The NPT ensemble was then annealed at a cooling rate of 70 K/ns to room temperature (300 K). Finally, the NPT ensemble was used to calculate for 5 ns, at 300 K and 1 atm. The internal energy was confirmed to stabilize at the same time as the density of the system became 1.1 g/cm^3^.

**Figure 1:**
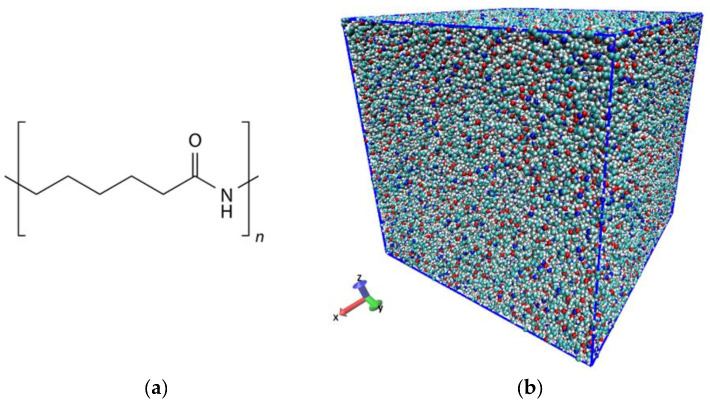
Illustration of (**a**) chemical structure of PA6, and (**b**) the initial system of PA6 consisting of 1000 molecules.

The initial temperature of the system was set to 300 K. A periodic boundary condition was used to reproduce the bulk system. The complete system is shown in Figure 1. The unit cell of the system was cubic, with sides measuring 17.4 mm.

MD simulations were performed using the GROMACS 2018.3 [34]. The OPLS-AA force field [35] was employed in this study and the parameters in the all-atom optimized potentials for liquid simulations (OPLS-AA) and electric state were determined using PolyParGen software [36]. To determine the ESP charge for each atom, the calculation level for structural stabilization was B3LYP/6-31G* and the electrification calculation was MP2/6-31G*. The long-range interaction was calculated using the particle mesh Ewald method [37] and the grid size was 0.12 nm. Van der Waals and Coulomb interactions were considered as non-bond interactions and the cutoff distance of the non-bond energy was 1.0 nm. The LINCS algorithm [38] was used to implement the MD simulations in time increments of 2 fs. These calculations were implemented using a cloud-based computer provided by Exabyte.io and GPU P100 (NVIDIA Corp., Santa Clara, CA, USA) supplied by Azure (Microsoft Corp., Albuquerque, NM, USA) [39].

The heat balance and strain energy methods were used to calculate the entropy generation. The equations based on the second law of thermodynamics, used for calculating the thermal entropy generation Δ*S* [J·s/K], are shown in Equations (1) and (2), respectively; the external work is calculated from the stress and displacement of the X-, Y-, and Z-planes while *T* is the temperature of the system at each instant in the MD simulations.

(1) $$\Delta S=\int_{t_{0}}^{t_{1}}\frac{\delta Q}{T}dt,$$

(2) δQ=δU−PδV,

Here, *Q*, *t*_1_, *t*_0_, *P*, *V*, *U*, and δ represent the heat quantity [J], time at fracture [s], initial time [s], pressure [Pa], volume [m^3^], internal energy [J], and time variation, respectively.

Next, the equations for calculating the entropy generation via the strain energy method (subsequently referred to as the mechanical entropy generation) $\Delta \gamma_{M}$ [J/K·m^3^] are shown in Equations (3) and (4) [9,32]. The dissipation strain energy was obtained by subtracting the elastic strain energy from the total strain energy. The dissipation strain energy is calculated for each X, Y, and Z plane and their total is the dissipation strain energy of the entire system. It should be noted that this equation can be used for experiments as well as MD.

(3) $$\Delta \gamma_{M}=\int_{0}^{t_{s}}\left(\frac{W_{p}}{T}\right)dt,$$

(4) $$W_{p}=W-W_{e},$$

where Δ*γ*_M_, *W*_p_, *W*, *W*_e_, and *t*_s_ represent the mechanical entropy generation [J/K·m^3^], inelastic strain energy [Pa], total strain energy [Pa], elastic strain energy [Pa], and time for an arbitrary strain [s], respectively. In this study, the evaluated strain before necking occurred was set to 0.25.

### 2.2. Simulation of Tensile Deformation

Tensile deformation simulations were performed using an isoenthalpic-isobaric (constant number of particles: N, pressure: P, enthalpy: H, as NPH) ensemble. To calculate the entropy generation, it was necessary to estimate the heat generated by the system owing to tension. In this study, the NPH ensemble was adopted so that the contribution of the heat bath to the total heat balance could be neglected. In the NPH ensemble, the X- and Y- directions were deformed via the Parinello–Rahman method [40] to maintain 1 atm and a uniaxial tension analysis was performed. Owing to the limitations of the simulation, the strain rate in the Z direction was assigned a constant value of 10^8^/s (engineering strain). However, the strain rate in the simulation was much higher than that in the experiment.

Tensile deformation simulation was also performed using GROMACS.2018.3, using the same simulation parameters as in the previous section. The initial temperature of the system was set to 300 K.

### 2.3. Results of MD Simulations

**Figure 2:**
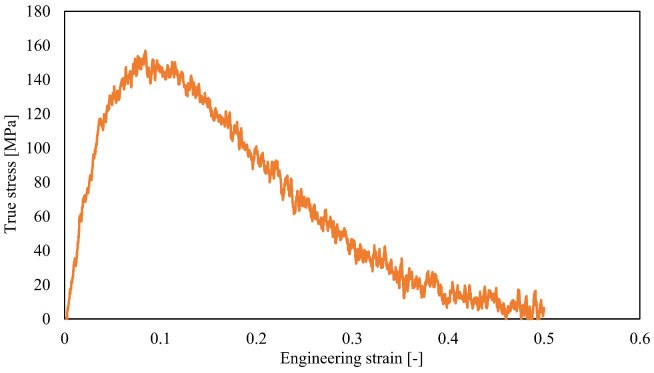
Stress–strain curve of PA6 obtained using MD simulations.

Figure 2 shows a typical stress-strain relationship. We observed that the stress increased with strain, reaching its maximum value (tensile strength) of 156 MPa at a strain of 0.1 before decreasing. The tensile strength in this study was higher than the experimental value because of its calculation volume [34] and the effect of strain rate on viscoelastic deformation.

**Figure 3:**
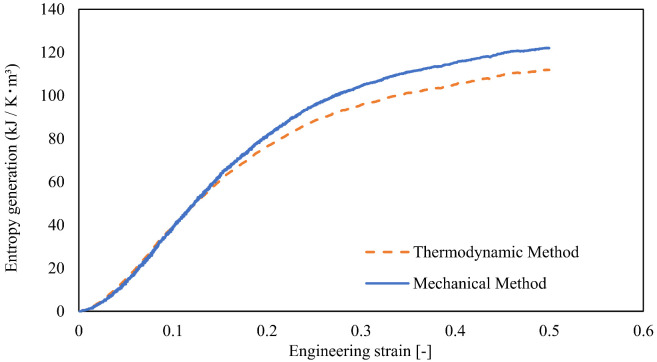
Fracture and thermal entropy generation of PA6 during tensile analysis using MD simulations.

We obtained the dissipation strain energy from the stress-strain relationship. The mechanical entropy generation, $\gamma_{M},$ was calculated using Equations (3) and (4), respectively: the external work was calculated using the stress data and displacement of each of the X-, Y-, and Z planes. The thermal entropy generation was obtained via the external work and Equations (1) and (2). The mechanical and thermal entropy generations are indicated by the solid and dotted lines, respectively, in Figure 3. Both entropy generations increase with strain, with almost the same value. In the high strain region, these entropy generations differ slightly because of the difference in the calculation method. When the strain is 0.25 (above which necking occurred during the experiments, as explained in the next section), the mechanical and thermal entropies are 95.3 and 88.2 kJ/K·m^3^, respectively.

## 3. Tensile Test Experiment

### 3.1. Materials and Methodology of the Tensile Test

**Figure 4:**
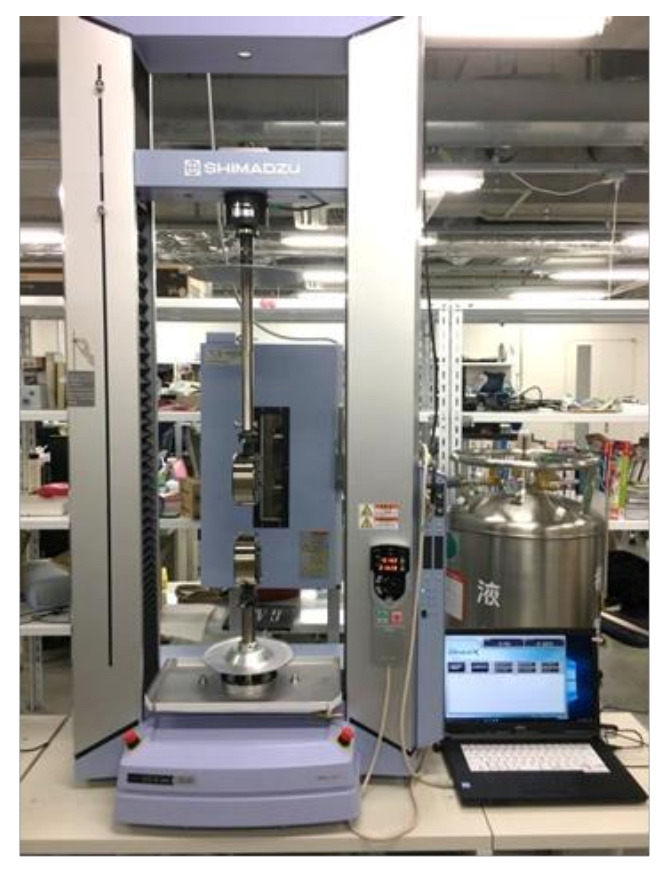
Universal mechanical testing machine (AG-X plus, SHIMADZU Co., Ltd., Kyoto, Japan) for conducting tensile tests.

As described in a previous paper [41], PA6 resin (CM1006, Toray Industries, Inc., Tokyo, Japan) was used for the experiments. Tensile tests were conducted using a universal mechanical testing machine (AG-X plus, Shimadzu Co., Ltd., Kyoto, Japan, shown in Figure 4) at room temperature, with a crosshead speed of 1 mm/min to confirm the results of the MD simulations.

### 3.2. Results of the Tensile Tests

**Figure 5:**
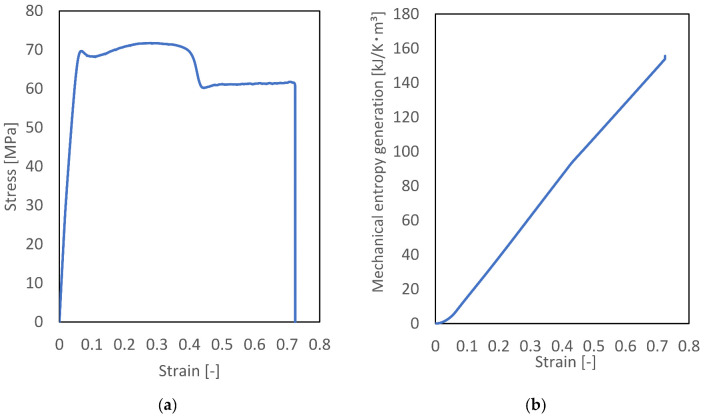
(**a**) Stress–strain diagram of PA6 obtained experimentally from the tensile test and (**b**) mechanical entropy generation, calculated using experimental data.

The results of the tensile tests are shown in Figure 5a. As the strain increases, the stress increases sharply and linearly. When the strain reached 0.07, the stress–strain curve exhibited a change from elastic to plastic behavior. At a strain of 0.25, the materials exhibited necking behavior and the stress remained constant. The maximum stress and Young’s modulus are 71.72 MPa and 3.66 GPa, respectively.

Mechanical entropy generation was calculated from the experimental stress-strain curve and Equations (3) and (4), respectively; Figure 5b shows the mechanical entropy generated by the tensile test. When the strain was 0.25, the mechanical entropy generation was 50.3 kJ/K·m^3^. The entropy generation obtained using MD was slightly higher owing to the scale difference; however, the experimentally and numerically obtained mechanical entropy generations were similar.

## 4. Thermal Entropy

### 4.1. Differential Scanning Calorimetry (DSC) Procedure

To determine the thermal entropy generation, the specific heat capacity was measured using a DSC-60 plus (Shimadzu Corp., Kyoto, Japan) with a stochastic temperature-modulated system. The heat flow and heat capacity were calibrated using indium and a sapphire standard, respectively. The samples were analyzed in crimped aluminum pans with ordinary lids. The reference was an empty pan of identical type as the sample pan and equal weight (matched within ±0.1 mg). A small amount of sample (~3 mg) was whittled from the surface of the samples before and after applying tensile deformation.

The measurements were conducted at a heating rate of 2 °C/min, temperature range of 0–45 °C, modulation period of 60 s, and amplitude of ±0.3 °C. The resultant heat flow between the sample and reference in the DSC experiment is described by the following [42]:

(5) $$\frac{dQ}{dt}=C_{p}b+f\left(T_{\mathrm{DSC}},t_{\mathrm{DSC}}\right),$$

Here, d*Q*/d*t* is the resultant heat flow, *T*_DSC_ is the temperature amplitude, *t*_DSC_ is the modulation period, *C*_p_ is the heat capacity of PA6 [J/K], *b* is the rate of temperature change (d*T*/d*t*), and *f*(*T*_DSC_, *t*_DSC_) is the heat flow from the kinetic process. The specific heat capacity, *c*_PS_, was obtained from the heat capacity and sample weight of the DSC.

### 4.2. Specific Heat Capacity

**Figure 6:**
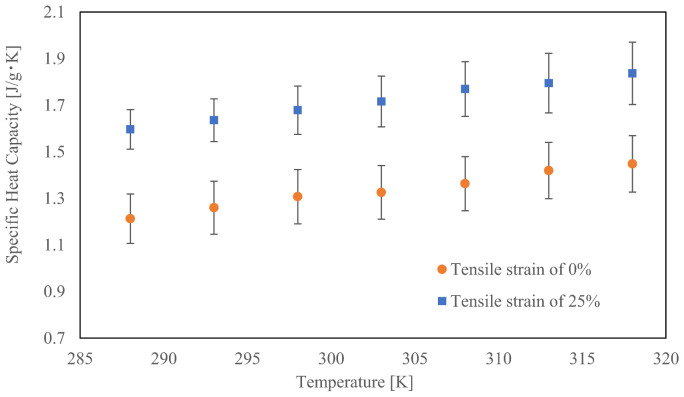
Relationship between the tensile strain and specific heat capacity.

Figure 6 shows the specific heat capacity of PA6, where each plot shows the averaged data of 15 samples and the error bar indicates the standard deviation. For the DSC measurements, the temperature ranged from 0 to 45 °C. However, owing to some deviation in the measurement results between 0 °C and 10 °C, we focused on the results temperatures ranging from 15 to 45 °C. As shown in this figure, the specific heat capacity *c*_PS_ increases linearly with temperature. The specific heat capacity of the tensile strain of 0% was lower than that of 25%, suggesting that the specific heat capacity depended on the tensile strain.

The thermal entropy generation Δ*S_T_* [J/K] was calculated using Equation (6).

(6) $$\Delta S_{T}=\int_{T_{0}}^{T_{1}}\frac{C_{pS}}{T}dT,$$

where *C*_pS_ is the heat capacity of PA6. In this study, we used *c*_pS_ instead of *C*_pS_ to calculate the specific thermal entropy generation Δ*s_T_* [J/K·g] for comparison with the mechanical entropy generation $\Delta \gamma_{M}$ [J/K·m^3^], as shown in Equation (5).

(7) $$\Delta s_{T}=\int_{T_{0}}^{T_{1}}\frac{c_{pS}}{T}dT$$

It is necessary to convert the unit of specific entropy to compare specific entropies. In this work, we used a density of 1130 g/cm^3^ for PA6 to convert Δ*s_T_* to entropy generation per unit volume.

### 4.3. Thermal Entropy Generation

**Figure 7:**
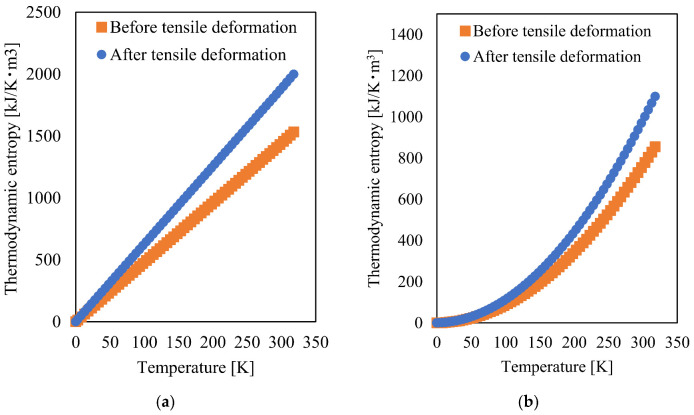
Thermalentropy generation before and after deformation, calculated using (**a**) linear and (**b**) quadratic extrapolation of the specific heat capacity.

To investigate the increase in specific thermal entropies associated with tensile deformation, we calculated them before and after tensile deformation using Equation (7), and the data obtained by extrapolating both capacities to 0 K. As the behavior of the thermal entropy generation of this material at room temperature was unknown, it was confirmed using two extrapolation methods: linear and quadratic, the results of which are shown in Figure 7a,b, respectively.

**Figure 8:**
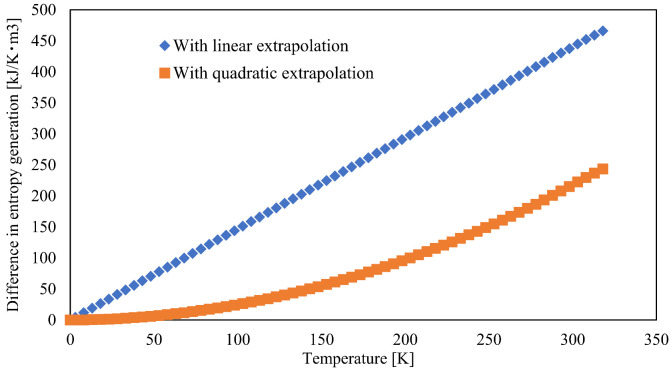
Thermalentropy generation before and after tensile deformation with linear and quadratic extrapolation.

As shown in Figure 7, the entropy generation before tensile deformation is lower than that after tensile deformation, following the same tendency as the specific heat capacities. The difference in specific thermal entropies before and after tensile deformation, calculated using linear and quadratic extrapolations, is shown in Figure 8. The differences in entropy generation in Figure 8 at room temperature are 437.5 kJ/K·m^3^ and 215.2 kJ/K·m^3^ for linear and quadratic extrapolation, respectively. These values are higher than the mechanical entropy generation obtained from MD and experiments. There are two possible reasons for this difference. First, there may be a problem in preparing the sample for DSC. DSC samples, whittled from the specimen surface by the same method, should have the same mechanical loading. Therefore, the value of the thermal entropy generation increases. For example, if both entropy values are twice the original values, the entropy generation is twice that of the original values. Thus, entropy generation may be overestimated. To solve this problem, a non-destructive method for measuring the heat capacity is desired, which is an interesting aspect for future investigations. Another limitation is that the extrapolation method for heat capacity may be inappropriate. In Figure 7, there is a difference between the two extrapolation methods, and consequently, a large difference is observed between the thermal entropy generation difference of the two extrapolation methods in Figure 8. Therefore, an appropriate extrapolation method for the heat capacity of PA6 is required to precisely determine the entropy. Although the entropy generation differs depending on the extrapolation method, its value increases with tensile deformation. Consequently, all entropy values obtained from MD, mechanical experiments, and DSC in this study increased qualitatively with mechanical loading. The measurement of entropy is a promising candidate for evaluating the invisible damage because it can identify the mechanical loading of materials using thermal entropy generation obtained by thermal analysis, such as DSC.

## 5. Conclusions

The relationship between the mechanical loading and entropies of PA6 (of which one was obtained from dissipative strain energy and the other from thermal entropy generation), was investigated under the same mechanical loading conditions.

During experimental testing and MD simulations, the mechanical entropy generation was calculated from the stress-strain relationship. In the MD simulations, the mechanical and thermal entropies were 95.3 kJ/K·m^3^ and 88.2 kJ/K·m^3^, respectively, when the strain was 0.25. During experimental testing, the mechanical entropy generation was 50.3 kJ/K·m^3^ at the same strain. The entropy generation obtained using MD was slightly higher because of the scale difference. However, the experimentally and numerically obtained mechanical entropy generation was similar.

Thermalentropy generation was calculated using the specific heat capacity measured by DSC via two extrapolation methods. The entropy values obtained by both methods were higher than those obtained via MD or experimentally and the thermal entropy obtained by MD. However, thermal entropy generation also increased as the applied tensile strain increased.

Consequently, it was suggested that thermal approaches, such as the specific heat capacity measured by DSC, could be used to directly measure and evaluate the invisible damage—i.e., free volume change, void and crack initiation, and damage—and the remaining life of materials. Although these entropy values are different in the present study, several possibilities exist for estimating the lower values obtained from DSC and MD and they would be closer values according to the procedures. The results of this study are the first to consider the deformation of polymeric materials from an entropic perspective and demonstrate the possibility of quantitative detection of mechanically induced invisible damage by measuring thermal properties via entropy generation of a polymer material. A quantitative comparison of the entropy values obtained from MD, DSC, and mechanical experiments will be included in our future work.

## Data Availability

Not applicable.
